# The impact of intraoperative ureteral stent omission on postoperative complications during radical cystectomy

**DOI:** 10.1007/s00345-026-06572-1

**Published:** 2026-08-21

**Authors:** John Pfail, Connor O’Leary, Melinda Fu, Alain Kaldany, Kevin Chua, Rachel Passarelli, Benjamin Lichtbroun, Hiren Patel, Arnav Srivastava, David Golombos, Thomas L. Jang, Vignesh T. Packiam, Saum Ghodoussipour

**Affiliations:** 1 Section of Urologic Oncology, Rutgers Cancer Institute and Rutgers Robert Wood Johnson Medical School, New Brunswick, NJ USA; 2 Department of Urology, The Ohio State University Wexner Medical Center, Columbus, OH USA; 3 Division of Urologic Oncology, University of Michigan Rogel Cancer Center, Ann Arbor, MI USA

**Keywords:** Urologic oncology, Ureteral stent, Postoperative complications, Radical cystectomy

## Abstract

**Introduction:**

Ureteral stents are traditionally placed intraoperatively during radical cystectomy (RC) to optimize healing and avoid short and long-term complications. However, strong evidence supporting this practice is lacking. We aim to assess the impact of ureteral stent placement with postoperative complications following RC.

**Methods:**

The National Surgical Quality Improvement Program database included 4,375 patients who underwent RC from 2019 to 2021. Baseline characteristics and complication rates were stratified by utilization of ureteral stenting. Primary endpoints included 30-day complications, length of stay (LOS), and readmissions. Multivariable analyses was performed to determine the association of no stent placement with postoperative morbidity.

**Results:**

Of the 4,375 patients who underwent RC, 413 (9.4%) did not receive intraoperative stent placement. Patients without stent placement had lower ASA (ASA < 3: 21% v. 27%; *p* < 0.01), lower median BMI 27 v. 28; *p* < 0.01), and lower median operative times (292 min v. 322; *p* < 0.01). There was no difference in preoperative median estimated glomerular filtration rate (CKD-EPI creatine Eq. 2021) between patients with or without stents placed (70 mL/min/1.73m^2^ v. 71 respectively; *p* = 0.8). Patients without stent placement had similar median LOS (6 days v. 6 days; *p* = 0.34) and readmission rates (23% v. 21%; *p* = 0.43) compared to those who had stents placed. After controlling for potential confounders, RC without stent placement was not associated with increased rates of any complication (OR: 1.23; 95% CI [0.99–1.51]), renal failure (OR: 1.4 [0.77–2.54]), ureteral stricture (OR: 0.6 [0.29–1.24]), urine leak (OR: 1.54 [0.91–2.62]), or UTI (OR: 1.18 [0.79–1.72]) at 30 days postoperatively.

**Conclusions:**

The omission of ureteral stents in a select population of patients did not appear to increase the risk of morbidity including 30-day complications, postoperative renal failure, UTI, urine leak, or ureteral strictures, indicating stentless RC may be performed safely in select patients.

**Supplementary Information:**

The online version contains supplementary material available at 10.1007/s00345-026-06572-1.

## Highlights

- Foregoing stent placement can be safe in select patients undergoing radical cystectomy.
- Omission of stent placement did not increase 30-day complication rates.
- Stentless RC was not associated with an increased LOS.

**Supplementary Information:**

The online version contains supplementary material available at 10.1007/s00345-026-06572-1.

## Introduction

With an estimated 84,870 new cases and 17,420 projected deaths in 2025, bladder cancer ranks as the sixth most prevalent cancer type in the United States [1]. Roughly 25% of individuals with bladder cancer are found to have muscle-invasive bladder cancer (MIBC) upon diagnosis [2]. For patients with MIBC, high-risk non-muscle invasive tumors, and in cases where intravesical or trimodal therapy has not been successful, radical cystectomy (RC) and urinary diversion serves as a standard-of-care treatment [3, 4]. Even with advancements over the last decade such as implementation of enhanced recover after surgery (ERAS) protocols, radical cystectomy remains a high-risk procedure and over half of patients undergoing RC experience complications within 30-days [5–7]. As RC represents the best choice for many patients with bladder cancer, there is a clear demand for evidence to reduce complication rates.

Ileal conduit and orthotopic neobladder are the most common forms of urinary diversion accompanying RC [8]. These procedures may be complicated by ureteroileal anastomosis stricture or urine leakage, which majorly impacts patients’ lives [9]. Many surgeons choose to place intraoperative ureteral stents during RC with urinary diversion operations with the rationale that the stent improves mechanical support, provides better alignment of the anastomosis, and may reduce the risk of ureteroenteric strictures [10]. In Skinner et al.’s series, which combined routine stent placement with a no-touch technique, the twelve-year stricture rate was only 2.6% [11]. Other surgeons, however, intentionally omit ureteral stents due to longer operative times with stent placement and perceived risk of a foreign body inflammatory reaction [12].

A recently published study by Shah et al. used the NSQIP database to compare outcomes between stented and stentless ileal conduits following radical cystectomy [13]. While their analysis provides valuable initial insights, it was limited to univariate comparisons without adjusting for potential confounders such as surgical approach, operative time, or comorbidities. Given that stent omission may be more common in patients with favorable baseline characteristics, the absence of multivariable adjustment may bias interpretation of safety. Our study addresses uses multivariable regression analyses to isolate the independent association of stent omission with early postoperative complications.

In this study, we aim to understand the correlation of intraoperative ureteral stent placement vs. stentless urinary diversion during RC procedures with 30-day morbidity. We hypothesize that stentless RC may have equivalent morbidity when compared to RC performed with the use of ureteral stent placement.

## Methods

### Patient selection

The American College of Surgeons’ National Surgical Quality Improvement Program (NSQIP) database, which includes 4351 patients who underwent an RC from 2019 to 2021, presents an opportunity to retrospectively study variables potentially relating to 30-day complication rates. Specifically, we used the NSQIP database’s targeted procedure-specific cystectomy files. Inclusion criteria included patients undergoing RC with either continent or incontinent diversion for urothelial carcinoma. Patients with systemic inflammatory response syndrome (SIRS), sepsis, or septic shock within 48 h prior to surgery were excluded to minimize the confounding from severe preoperative infection that could independently impact postoperative morbidity. Since all the data in NSQIP is deidentified, this study was exempt from review at our institutional review board (Study ID: Pro2023000635).

We identified the procedures patients underwent via common procedural terminology (CPT) codes. Procedures included from the cystectomy file were complete cystectomy (51595, 51596, 51590, 51570, 51597, 51575, 51585, and 51565). All patients were stratified by intraoperative stent placement (i.e., “Stentless” vs. “Stent” cohorts).

### Variables

We captured demographic variables – including age and sex – and clinical variables including comorbidity data. Estimated glomerular filtration rate (eGFR) was calculated using the CKD-EPI 2021 creatine equation using preoperative creatinine values recorded in NSQIP [14]. Pathologic T, N, and M staging was also extracted from the targeted datasets. Using this data, patients were classified according to the American Joint Committee on Cancer (AJCC) staging. As per the NSQIP definition, preoperative chemotherapy was defined as any systemic chemotherapy received within 90-days preoperatively.

Operative characteristics included operative time, urinary diversion type (incontinent or continent), and surgical approach (open, hybrid, or laparoscopic/robotic). Postoperative complications at 30-days were collected and categorized as either low or high grade (Supplemental Table 1). Management approach rather than diagnostic label determined high- vs. low-grade assignment. RC specific complications included ureteral obstruction/stricture, defined as new onset or worsening hydronephrosis diagnosed with either sonogram, intravenous pyelogram, or CT, renal failure, defined in the NSQIP dataset as a rise in creatinine of > 2 mg/dl from preoperative value or need for new dialysis and urine leak defined as persistent drainage (on or after POD#3) which is suggestive of or diagnosed as a urine leak or fistula including presence of creatine-rich fluid (creatinine content greater than 3 times serum creatinine) and one of the following: drain continued longer than 7 days, spontaneous wound drainage, percutaneous drainage performed, endoscopic intervention, reoperation performed. Within the 30-day follow-up period of the NSQIP dataset, length of stay, readmission rates, and mortality were also investigated. Patients with missing data for any listed variable were excluded from the analysis a priori to ensure data completeness and consistency across comparisons.

### Statistical analysis

Chi-squared test was used for nonparametric categorical variables and the Mann-Whitney U test was used for nonparametric continuous variables. Univariable and multivariable logistic regression was performed for 30-day complications. Additionally, a univariable and multivariable linear regression was performed for length of stay (LOS). Variables included age, ASA score, BMI, surgical approach (open vs. hybrid vs. laparoscopic/robotic), prior chemotherapy, prior pelvic radiation, operation time, intraoperative stent placement, pathologic stage group, and urinary diversion type (incontinent vs. continent diversion). The NSQIP dataset does not provide the Charlson comorbidity index so we adjusted for relevant comorbidities and included ASA index as a proxy for perioperative risk. In the regression analyses, any patient with missing data was not included in the analysis. All variables were considered in univariable analyses. Factors included in the multivariable analysis included those that were clinically relevant (age, BMI, ASA, operation time, pathologic stage group, and stent placement) or had a p-value less than 0.05 on univariable analysis. On the final multivariable analyses, covariates included, age, diversion type (incontinent vs. continent), approach (open vs. hybrid vs. robotic/laparoscopic), ASA, BMI, prior chemotherapy, prior pelvic surgery, operative time, and AJCC stage. Our primary endpoint was overall complication rate (tested at α = 0.05). Secondary endpoints—low-grade complication, high-grade complication, ureteral obstruction, lymphocele/other fluid collection, urine leak, renal failure, UTI, LOS, and readmission—were considered hypothesis-generating; their p-values are reported nominally without adjustment for multiplicity. All analyses were conducted using R (R foundation, Vienna) version 4.1.2.

## Results

We identified 4,375 patients who underwent radical cystectomy with urinary diversion, all of whom had data available regarding intraoperative stent placement. Stentless RC was performed in 9% (413/4375) of patients undergoing RC (Table 1). Patients who underwent RC without stent placement were more likely to undergo a robotic/laparoscopic approach (35.8% vs. 22.3%, *p* < 0.001), have a lower ASA score (< 3: 26.6% vs. 20.9%, *p* = 0.08) and BMI (median BMI: 27.0 vs. 27.8, *p* = 0.004), lower rates of prior chemotherapy (29.8% vs. 37.2%, *p* = 0.004), higher rates of prior pelvic surgery (55.7% vs. 47.7%, *p* = 0.002), and shorter operative time (median OR time: 292 min vs. 322, *p* < 0.001) compared to those who had a stent placed, respectively. There were no significant differences in age, sex, diversion type, diabetes, smoking history, chronic obstructive pulmonary disease (COPD), congestive heart failure (CHF), eGFR, prior pelvic radiation, or AJCC stage.

**Table 1:** Preoperative and operative characteristics for patients undergoing RC, stratified by stent placement

	Stentless	Stent	*p*-value
	*N* = 413 (9%)	*N* = 3962 (91%)	
Diversion:			0.314
Incontinent	364 (88.1%)	3416 (86.2%)	
Continent	49 (11.9%)	546 (13.8%)	
Approach:			**<0.001**
Open	260 (63.0%)	2879 (72.7%)	
Hybrid	5 (1.21%)	200 (5.05%)	
Laparoscopic/Robotic	148 (35.8%)	883 (22.3%)	
Age	70.0 [64.0;76.0]	71.0 [64.0;76.0]	0.627
Sex:			0.756
Female	74 (17.9%)	740 (18.7%)	
Male	339 (82.1%)	3222 (81.3%)	
ASA:			**0.008**
<3	110 (26.6%)	827 (20.9%)	
≥ 3	303 (73.4%)	3135 (79.1%)	
BMI	27.0 [24.1;30.6]	27.8 [24.8;31.7]	**0.004**
Diabetes	81 (19.6%)	764 (19.3%)	0.924
Smoking Hx	87 (21.1%)	826 (20.8%)	0.968
COPD	32 (7.75%)	249 (6.28%)	0.294
CHF	6 (1.45%)	44 (1.11%)	0.468
eGFR	70.6 [51.4;89.0]	70.0 [53.4;88.0]	0.796
Prior Chemotherapy:	123 (29.8%)	1473 (37.2%)	**0.004**
Prior Pelvic Radiation:	28 (6.78%)	340 (8.58%)	0.245
Prior Pelvic Surgery:	230 (55.7%)	1891 (47.7%)	**0.002**
OR Time:	292 [218;381]	322 [249;405]	**<0.001**
AJCC Stage:			0.874
0a-II	210 (50.8%)	1993 (50.3%)	
III-IV	203 (49.2%)	1969 (49.7%)	

Age and operating room (OR) time presented as median [interquartile range (IQR)]. Patients with missing data for any listed variable were excluded from the analysis.

In patients undergoing RC, omission of stent was not associated with an increased risk of any complication, high grade complications or procedure specific complications (Table 2). Furthermore, there was no difference in hospital length of stay (median: 6 days vs. 6 days, *p* = 0.434), 30-day readmission (22.8% vs. 21.0%, *p* = 0.434) or mortality (1.45% vs. 1.67%, *p* = 0.904) rates.

**Table 2:** 30-day complication rates for patients undergoing RC, stratified by stent placement

Outcome	Stentless	Stent	*p*-value
	*N* = 413 (9%)	*N* = 3962 (91%)	
Any Complication:	190 (46.0%)	1690 (42.7%)	0.209
High Grade:	75 (18.2%)	663 (16.7%)	0.505
Low Grade:	97 (23.5%)	871 (22.0%)	0.524
Ureteral Obstruction:	8 (1.94%)	127 (3.21%)	0.204
UTI:	31 (7.51%)	250 (6.31%)	0.402
Lymphocele / Other Fluid:	23 (5.57%)	225 (5.68%)	1
Urine Leak:	17 (4.12%)	126 (3.18%)	0.383
Renal Failure:	13 (3.15%)	107 (2.70%)	0.711
Reoperation:	18 (4.36%)	151 (3.81%)	0.678
LOS:	6.00 [5.00;8.00]	6.00 [5.00;8.00]	0.335
Readmission:	94 (22.8%)	831 (21.0%)	0.434
Mortality:	6 (1.45%)	66 (1.67%)	0.904

UTI = Urinary Tract Infection; LOS = Length of Stay, median [IQR]

**Fig. 1:**
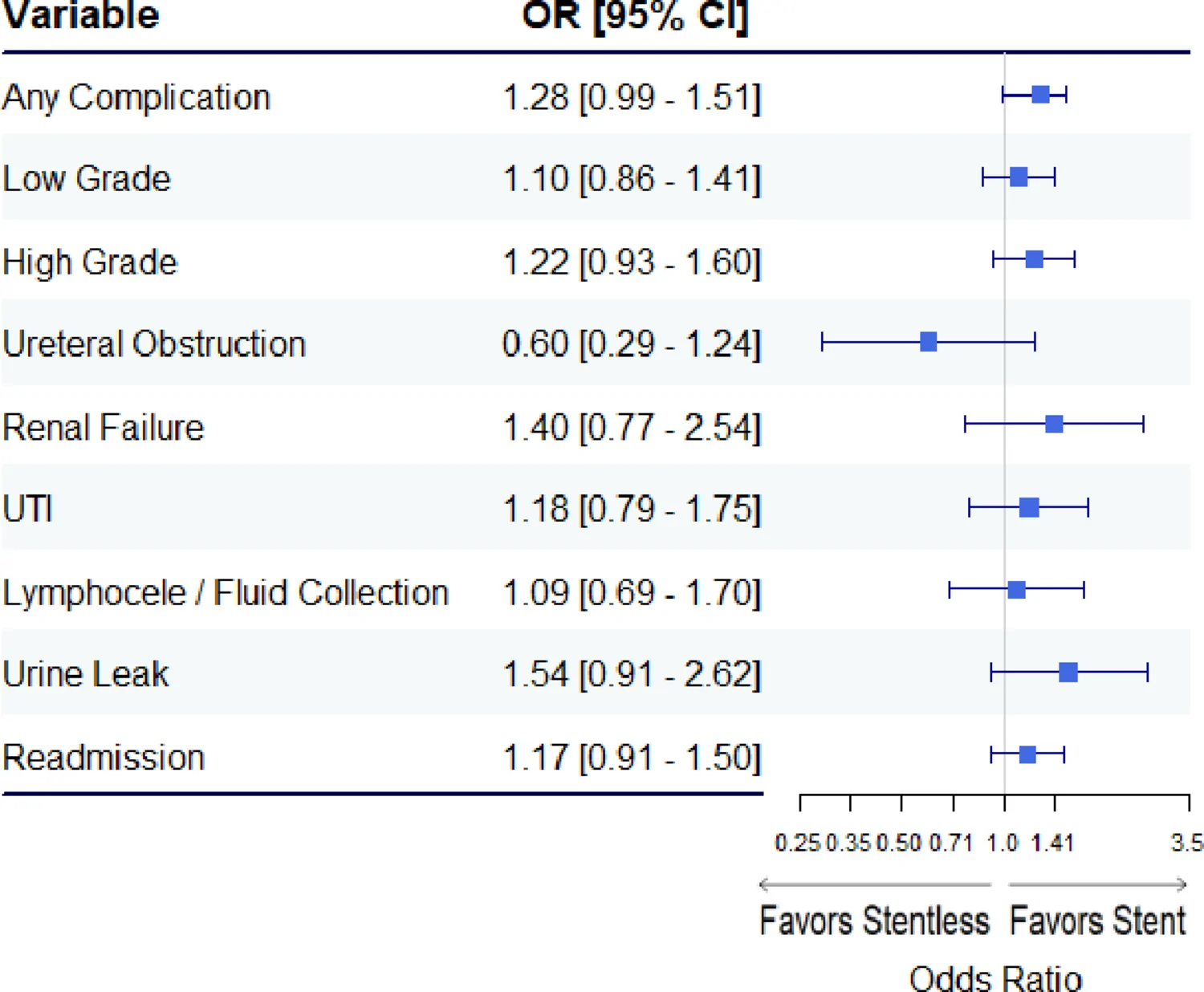
Odds ratios (OR) [95% CI] assessing the risk of complications associated with stent placement. Covariates included age, diversion type (incontinent vs. continent), stent placement, approach (open vs. hybrid vs. robotic/laparoscopic), ASA, BMI, prior chemotherapy, prior pelvic surgery, operative time, and AJCC stage. *Reference = Stent placed*

On multivariable analysis, the omission of stent placement was not associated with increased odds of any complication (OR: 1.28 [0.99–1.51]) or high-grade complications (OR:1.22 [ 0.93–1.60]) when compared to those who underwent RC with stent placement (Fig. 1). Similarly, there was no significant difference in the odds of RC specific complications including ureteral obstruction (OR: 0.6 [0.29–1.24]), renal failure (OR: 1.4 [0.77–2.54]), UTI (OR: 1.18 [0.79–1.75]), lymphocele / fluid collection (OR: 1.09 [0.69–1.70]), urine leak (OR: 1.54 [0.91–2.62]), or readmission (OR: 1.17 [0.91–1.50]).

Furthermore, we conducted an exploratory subgroup analysis between patients undergoing robotic vs. open radical cystectomy. In the open group, 92 out of 3,031 patients (3.0%) developed new ureteral obstruction, compared to 43 out of 1,185 patients (3.6%) in the robotic group. A chi-square test demonstrated no statistically significant difference between approaches (*p* ≈ 0.33), and the relative risk estimate suggested no clinically meaningful difference between open and robotic diversion techniques with respect to early ureteral stricture formation.

## Discussion

Our study leveraged a national cohort of over 4,000 patients who underwent either open or robot-assisted RC to characterize the association between the intentional omission of ureteral stents and complication rates. We found a stentless approach in a select group of patients was associated with decreased operative times without difference in LOS, readmission rates, rates of renal failure, ureteral stricture, UTI, urine leak, or other complications. Interestingly, although the stentless group had a higher proportion of patients with prior pelvic surgery, which could increase operative complexity and the risk of anastomotic complications, these patients did not demonstrate worse outcomes. This may reflect intraoperative selection by surgeons who judged stent omission safe despite prior surgical history, emphasizing the importance of surgeon experience and selection bias in interpreting our findings.

Our study extends upon prior work by Shah et al. by offering a more rigorous, adjusted analysis of the safety of stentless RC [13]. While Shah et al. reported similar rates of complications between stented and stentless groups, they did not control for important confounding variables such as ASA score, BMI, surgical approach (robotic vs. open), or prior treatments. Our multivariable models accounted for these covariates, strengthening the inference that ureteral stent omission is not independently associated with increased morbidity. Our study also categorizes complications by severity and provides odds ratios with confidence intervals to provide better clinical interpretation of the analysis. These methodological differences add important value to the existing literature and support the feasibility of selective stent omission during RC. If there is no increased risk of complication during a stentless RC, the intentional omission of a ureteral stent may lead to better outcomes by reducing operative time and the risk of foreign body contamination [12, 15]. A stentless approach also has potential to improve outcomes due to decreased mechanical injury from traction on the distal ureter [16–19].

The ERAS guidelines for radical cystectomy were developed with the goal of reducing complications and length of hospital stay after RC, a procedure that poses significant surgical challenges and morbidity [20]. The current guidelines recommend use of intraoperative ureteral stents for at least 5-days postoperatively (optimal duration of ureteral stenting unknown) [20] since stenting may be associated with better upper urinary tract drainage, improved bowel recovery, and decreased frequency of metabolic acidosis [16]. However, in its recommendations regarding the use of intraoperative stenting, the ERAS Society cited only a single study, Mattei et al., to support urinary drainage guidance. Thus, the qualification of the ERAS guidelines to recommend use of ureteral stents with “Very Low/Low” evidence for cystectomy/rectal surgery and a “Weak” grade of recommendation [20].

In the study by Mattei et al., 54 patients were randomized to undergo RC with intraoperative stents placed during the uretero-ileal anastomosis and immediately removed after completion (*n* = 25) while another group had the stent removed between postoperative day 5–10 (*n* = 29). This is in contrast to contemporary series on stentless RC where stents are typically omitted altogether rather than placement and removal immediately after the anastomosis [19, 21–24]. Mattei et al. found improved urinary drainage (increase of pelvicaliceal dilatation on POD#3: 16% vs. 40%; *p* = 0.025), bowel recovery (patients with flatulence on POD#3: 72% vs. 32%; *p* < 0.001), and decreased metabolic acidosis (determined by base excess values on POD#3: +1.9 vs. -1.0 mmol/l) in those who had stents placed for 5–10 days. Interestingly, they reported that 3 patients (10%) with stents left in during the perioperative period required anastomotic revision due to stricture while no patients in the immediate stent removal group required revision. They hypothesized that stricture formation was due to mechanical irritation from removing the stent during the anastomotic healing process. Another suggested etiology was traumatic injury to the distal ureter from traction of the stent during removal [16]. Evidence directly supporting the mechanism of stent removal theoretically causing distal ureteral trauma is limited and largely extrapolated from small series or analogies to other urologic procedures [25]. In other contexts, such as ureteroscopy, stent placement and removal are not consistently linked to clinically significant stricture formation [26]. Therefore, while the concept of stent-associated trauma is plausible, the true impact on ureteroenteric anastomotic stricture rates remains unclear and likely multifactorial, depending on factors such as tissue handling, ischemia, and surgical technique [17]. However, the absence of a truly stentless group in the Mattei et al. study combined with a small sample size limits definitive conclusions regarding association between ureteral stents and patient outcomes following RC.

The evidence regarding the benefits of ureteral stent use is inconclusive and recent research may support a stentless RC approach. Donat et al. found that despite the convention of using a ureteral stent during RC with urinary diversion, intraoperative stent use was associated with significantly higher infectious complications, urgent care visits, ureteral obstruction, and significantly increased odds of 30-day ureteroenteric anastomosis associated complications [18]. Numerous recent case cohort and retrospective studies have demonstrated the safety and feasibility of stentless RCs, albeit typically analyzing single-institution or single-surgeon patient samples [21–24]. In spite of the intended purpose of ureteral stents, a meta-analysis published by Peng et al. showed that stent use during RC did not reduce the rate of ureteroileal anastomotic stricture [17]. Further, a study by Ramahi et al. suggests that placement of ureteral stents may be associated with formation of ureteroenteric strictures [19]. This study reported a 19% ureteroenteric stricture rate at five years in an early series of entirely robotic radical cystectomies, substantially higher than the roughly 2.5% rate seen in more mature open cohorts with median follow up of 12.4 years [11], suggesting their findings likely reflect a learning-curve effect more than the intrinsic risk of stent placement. More recently, Tallman et al. reported that ureteroenteric anastomosis complications were higher in the stented group (20% vs. 9.5%) [27]. Similarly, Mahmood et al. found 7% and 10% stricture rates at one and two years in their stent-free group, again markedly higher than expected, raising concerns that single-center technique or selection bias may be driving these results [28]. While recent evidence suggests potential benefits with the use of a stentless approach at RC, these studies need to be supported by additional analyses with larger patient samples from multiple institutions to more broadly support intentional omission of ureteral stents during RC with urinary diversion.

While our results suggest that omission of ureteral stents may be safe in selected patients, it is also important to acknowledge the practical benefits that continue to favor stent placement for many surgeons. Ureteral stents are relatively low-cost, simple to insert intraoperatively, and typically remain in place for only a short period under normal recovery. Moreover, secondary stent placement or interventional radiology drainage in the event of a postoperative urine leak or stricture can add patient discomfort, procedural risks, and healthcare costs [29]. Thus, for many surgeons, routine stenting remains an effective and pragmatic measure to support safe anastomotic healing and minimize the need for additional postoperative interventions.

While stent omission was not statistically associated with increased urine leak, the confidence interval for this estimate was wide (OR 1.54; 95% CI 0.91–2.62), reflecting limited precision for relatively uncommon events in the stentless cohort. As such, moderate increases in risk cannot be definitively excluded.

The findings presented in our study should be considered in the context of several limitations. Our analysis is largely limited by restrictions inherent to using a retrospective national cohort database such as NSQIP, such as history of pelvic radiation, ureteral disease or involvement. Outcomes were recorded up to a limited time point of 30 days postoperative, which is not sufficiently long enough to capture the relationship between stent use and frequency of anastomotic stricture [9, 19]. While we have attempted to control for all relevant confounding variables, potential confounders not reported in the NSQIP database variables may exist. Inconsistency in reporting and data collection may compromise the quality of the data. Participating in NSQIP is optional, exposing this data to further selection bias. We cannot ascertain why surgeons chose to omit or place a stent, which introduces potential selection bias. NSQIP is designed to ensure 30-day follow up for all included patients, however, individual-level follow-up completeness is not identifiable in the dataset, and loss to follow-up could not be quantified. Furthermore, the data regarding stent placement was limited and we could not ascertain if the stentless cohort was completely stentless or the total duration or stent type placement in the stented cohort. The stented group is substantially larger, which may introduce imbalance and limit power for subgroup or matched analyses. Future analyses could consider matched cohorts, though the small proportion of stentless cases limited this in our analysis. Despite these limitations, our study is one of the largest single-intervention analysis focused on understanding the relationship between a stentless surgical approach and 30-day complications following radical cystectomy.

## Conclusion

Analysis of a large contemporary database evaluating the omission of intraoperative ureteral stent placement during RC did not show a significant increase in 30-day complications, LOS, or ureteral anastomotic related complications including renal failure, UTI, urine leak or ureteral strictures. Our findings suggest omission may be safe in select patients with lower comorbidity, lower operative complexity, or in experienced centers. However, clear selection criteria cannot be defined from this study alone. Further prospective or randomized studies are needed to better evaluate the risks/benefits of a stentless approach to RC and to determine if there are patient or clinical related factors that might be important for patient selection that are not apparent in large retrospective national cohort databases.

## Supplementary Information

Below is the link to the electronic supplementary material.

Supplementary Material 1.

## Data Availability

All data used is readily available via NSQIP.
