# Supplementary material for: The impact of intraoperative ureteral stent omission on postoperative complications during radical cystectomy
Source: World J Urol. 2026 Aug 21;44(1):593. doi: 10.1007/s00345-026-06572-1 (PMC13498471; doi:10.1007/s00345-026-06572-1)
Supplement: Supplementary file 1 [file 345_2026_6572_MOESM1_ESM.docx]

| Low Grade | Occurrences of Surgical Site Infection (SSI), deep incisional SSI, organ space SSI, fascial dehiscence, pneumonia, UTI, DVT, ileus (prolonged NPO/nasogastric tube), rectal injury identified postoperatively and not requiring intervention/surgery, C. diff, ureteral fistula (no operative intervention), ureteral obstruction (no operative intervention), lymphocele (no intervention), anastomotic leak (no intervention), progressive renal insufficiency |
| --- | --- |
|  | Unplanned reoperation, leak treated with intervention or reoperation, lymphocele with reoperation or intervention, ureteral obstruction requiring intervention |
| High Grade | Septic shock*, PE, MI, cardiac arrest necessitating CPR, unplanned intubation, ventilator dependent > 48 hours, acute renal failure necessitating dialysis, stroke |
|  | Patient death |

**Supplemental Table 1.** Grading definitions for postoperative complications.

*We excluded preoperative sepsis but captured postoperative sepsis requiring ICU care as a high-grade complication
